# Supplementary figures and images for: Macrophage network dynamics depend on haptokinesis for optimal local surveillance
Source: eLife. 2022 Mar 28;11:e75354. doi: 10.7554/eLife.75354 (PMC8963880; doi:10.7554/eLife.75354)

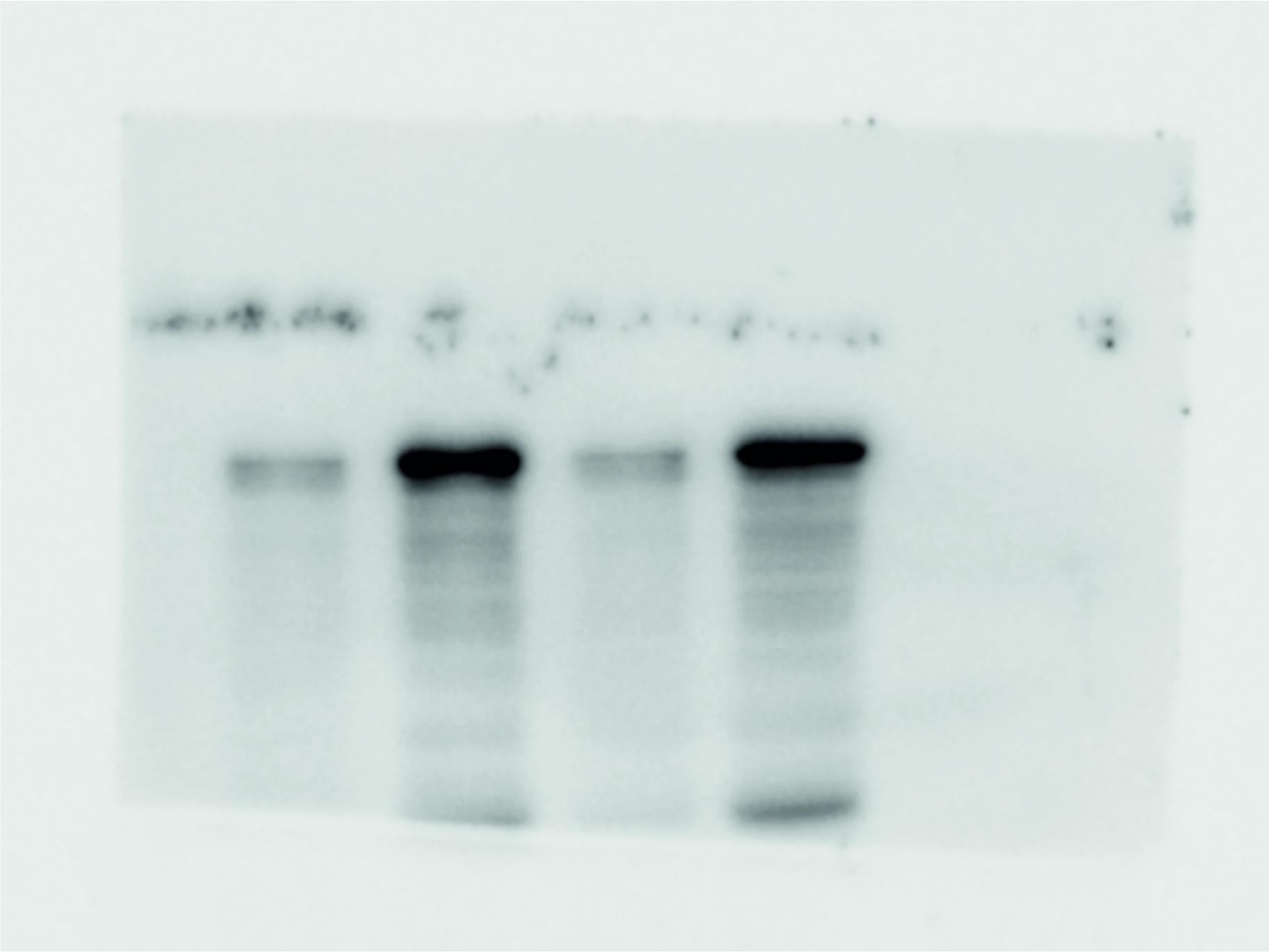

Supplement: Figure 1—figure supplement 2—source data 1. [file elife-75354-fig1-figsupp2-data1.zip › Talin.tif]

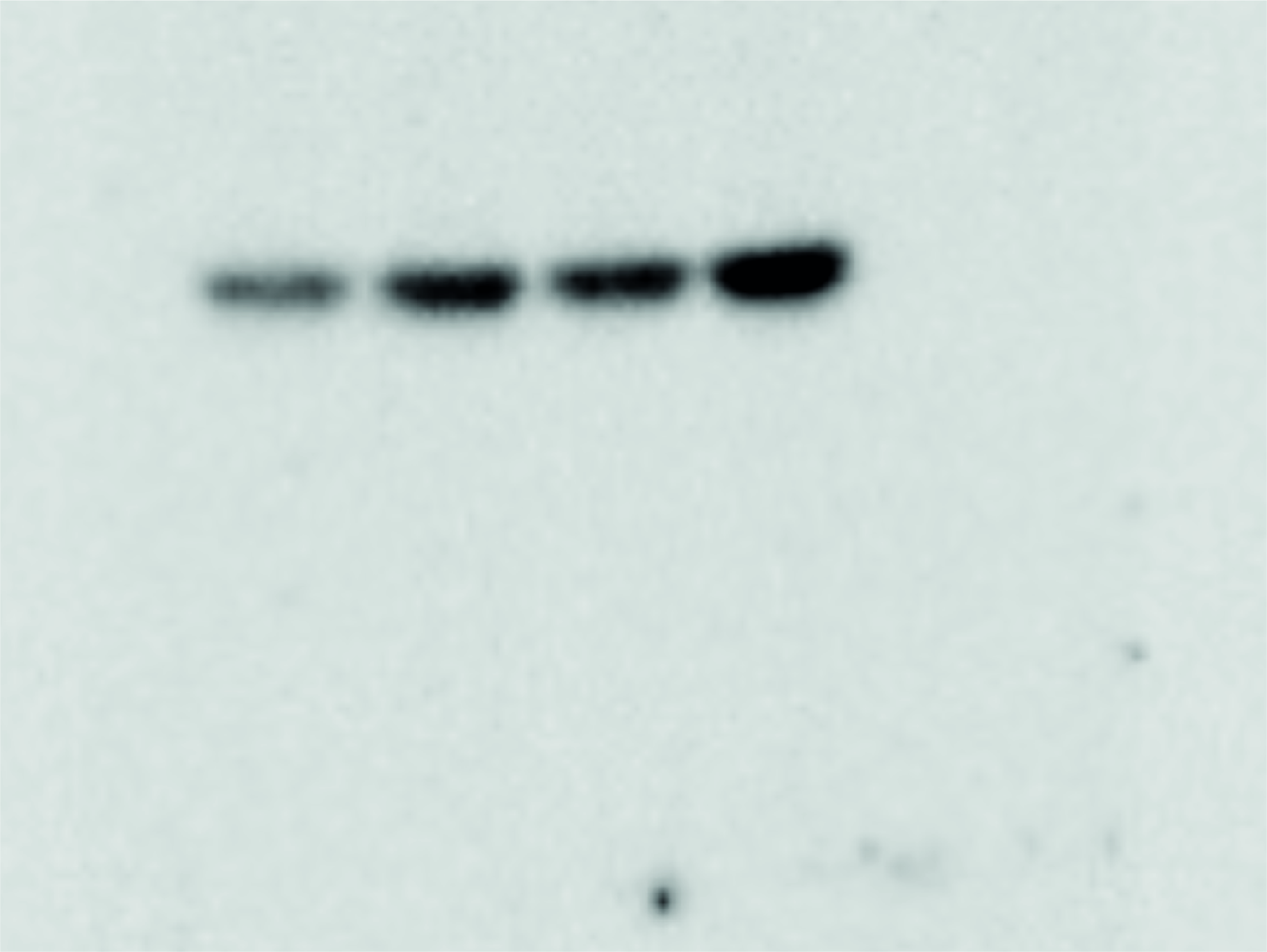

Supplement: Figure 1—figure supplement 2—source data 1. [file elife-75354-fig1-figsupp2-data1.zip › Actin.tif]

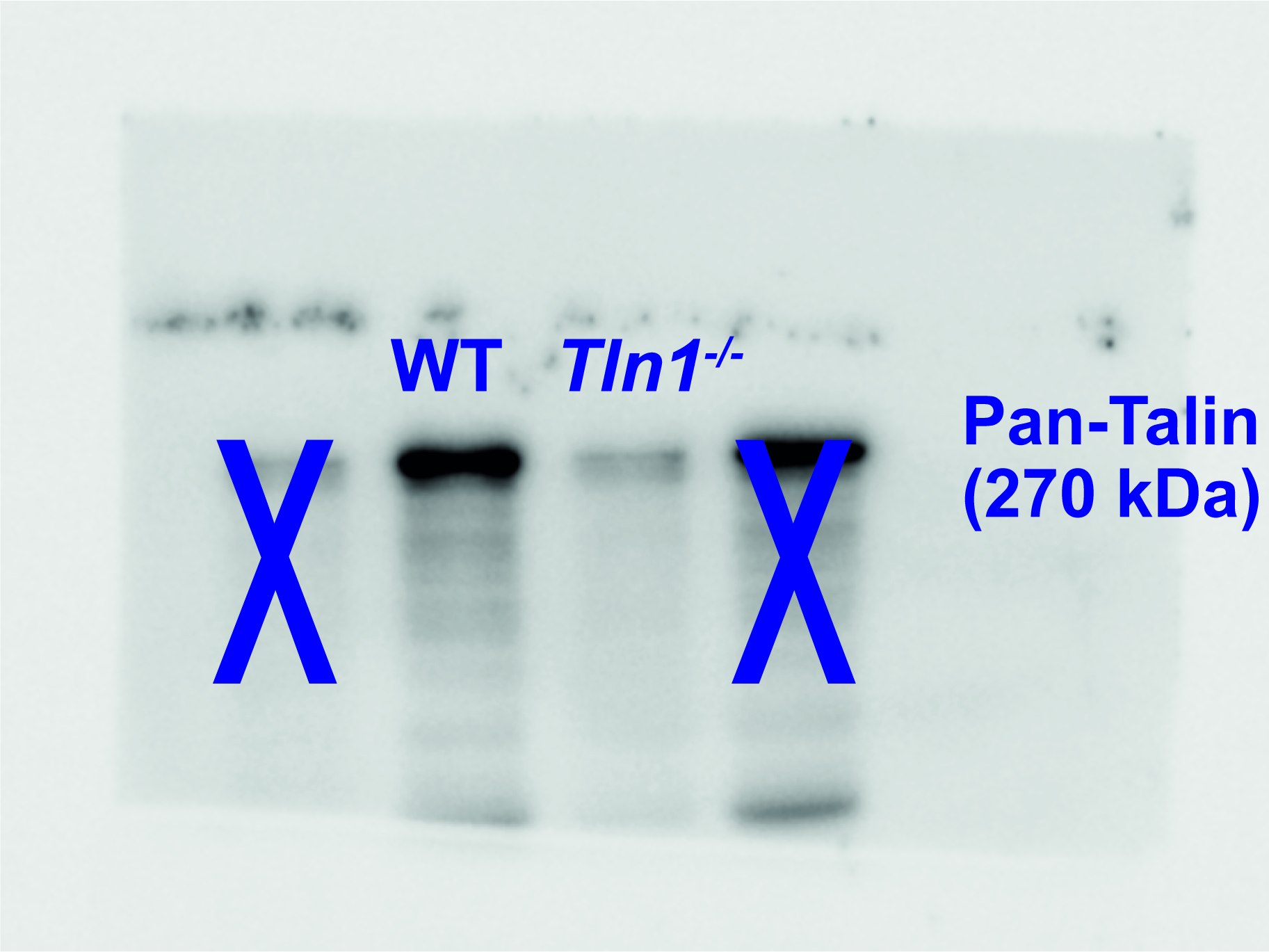

Supplement: Figure 1—figure supplement 2—source data 2. [file elife-75354-fig1-figsupp2-data2.zip › Talin annotated.tif]

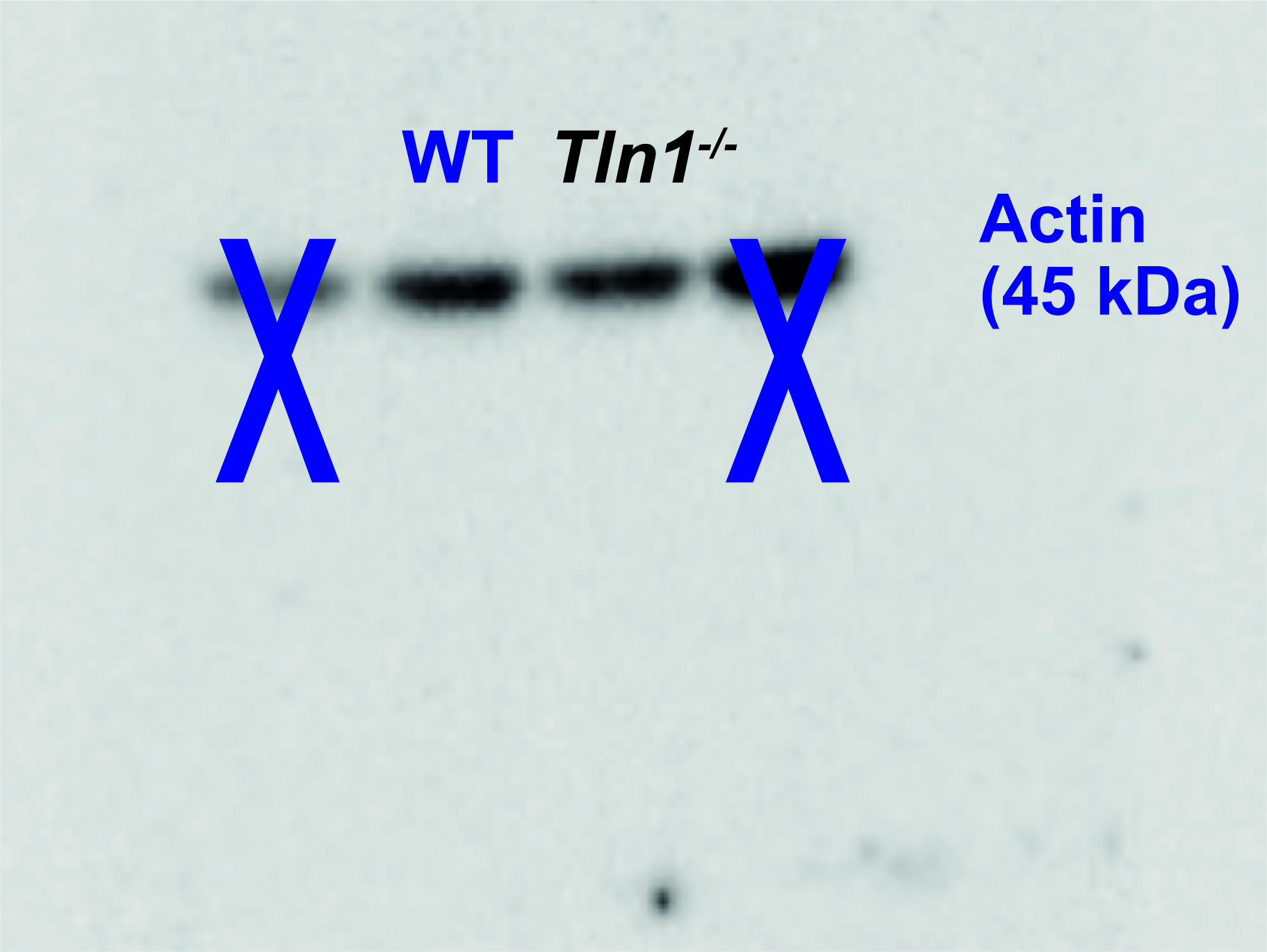

Supplement: Figure 1—figure supplement 2—source data 2. [file elife-75354-fig1-figsupp2-data2.zip › Actin annotated.tif]
