# Supplementary material for: Macrophage network dynamics depend on haptokinesis for optimal local surveillance
Source: eLife. 2022 Mar 28;11:e75354. doi: 10.7554/eLife.75354 (PMC8963880; doi:10.7554/eLife.75354)
Supplement: Supplementary file 1. [file elife-75354-supp1.docx]

**Supplementary File 1**

Table for overview on all statistical tests performed.

| **Figure** | **Statist. test** |  | **Posthoc test** |  |
| --- | --- | --- | --- | --- |
| 1D | **Kruskal-Wallis** | H=61.4, n(group)=3, P≤0.0001  N(Ctrl:CytoD)=25,25, N(Ctrl:Y27)=25,25 | **Dunn’s**  Control:CytoD  Control:Y27 | *** (P≤0.001)  *** (P≤0.001) |
| 1E | **ANOVA** | F(2, 6)=172.0, P≤0.0001 | **Dunnett’s**  Control:CytoD  Control:Y27 | *** (P≤ 0.001)  *** (P≤0.001) |
| 1G | **Kruskal-Wallis** | H=70.62, n(group)=4, P≤0.0001  N(WT: Tln1^-/-^)=25,25,  N(WT: Itgb2^-/-^)=25,25,  N(WT: Itgb1^-/-^)=25,25 | **Dunn’s**  WT: Tln1^-/-^  WT: Itgb2^-/-^  WT: Itgb1^-/-^ | *** (P≤0.001)  ns (P>0.05)  *** (P≤0.001) |
| 1H | **ANOVA** | F(3,8)=58.43, P≤0.0001 | **Dunnett’s**  WT: Tln1^-/-^  WT: Itgb2^-/-^  WT: Itgb1^-/-^ | *** (P≤0.001)  ns (P>0.05)  *** (P≤0.001) |
| 1 – fig suppl 1B | **unpaired two-tailed**  ***t* test** | t=9.993, df=48, P≤ 0.0001 | - | *** (P≤0.001) |
| 1 – fig suppl 1E | **Mann Whitn. *U* test** | U=40, N(Ctrl)=25, N(CK-666)=55,  P≤ 0.0001 | **-** | *** (P≤0.001) |
| 3D | **Kruskal-Wallis** | H=62.26, n(group)=3, P≤0.0001  N(Ctrl:CytoD)=25,25, N(Ctrl:Y27)=25,25 | **Dunn’s**  Control:CytoD  Control:Y27 | *** (P≤0.001)  *** (P≤0.001) |
| 3E | **ANOVA** | F(2,6)=848.8, P≤0.0001 | **Dunnett’s**  Control:CytoD  Control:Y27 | *** (P≤0.001)  *** (P≤0.001) |
| 3F | **ANOVA** | F(3,96)=1.025, P=0.3851  N(WT: Tln1^-/-^)=25,25,  N(WT: Itgb2^-/-^)=25,25,  N(WT: Itgb1^-/-^)=25,25 | **Dunnett’s**  WT: Tln1^-/-^  WT: Itgb2^-/-^  WT: Itgb1^-/-^ | ns (P>0.05)  ns (P>0.05)  ns (P>0.05) |
| 3G | **ANOVA** | F(3,8)=0.5293, P=0.6746 | **Dunnett’s**  WT: Tln1^-/-^  WT: Itgb2^-/-^  WT: Itgb1^-/-^ | ns (P>0.05)  ns (P>0.05)  ns (P>0.05) |
| 3 – fig suppl 1 | **unpaired two-tailed**  ***t* test** | t=1.116, df=48, P=0.27 | - | ns (P>0.05) |
| 3 – fig suppl 2A | **ANOVA** | F(3,8)=2.926, P=0.0998 | **Dunnett’s**  WT: Tln1^-/-^  WT: Itgb2^-/-^  WT: Itgb1^-/-^ | ns (P>0.05)  ns (P>0.05)  ns (P>0.05) |
| 3 – fig suppl 2C | **unpaired two-tailed**  ***t* test** | t=6.643, df=48, P≤ 0.0001 | - | *** (P≤0.001) |
| 4D | **Mann Whitn. *U* test** | U=871, N(WT)=37, N(Itgb1^-/-^)=55,  P=0.2455 | - | ns (P>0.05) |
| 5E | **ANOVA** | F(2,6)=418.8, P≤0.0001 | **Dunnett’s**  Control:CytoD  Control:Y27 | *** (P≤0.001)  ns (P>0.05) |
| 6C | **ANOVA** | F(3,8)=37.13, P≤0.0001 | **Dunnett’s**  WT: Tln1^-/-^  WT: Itgb2^-/-^  WT: Itgb1^-/-^ | *** (P≤0.001)  ns (P>0.05)  *** (P≤0.001) |
| 6F | **ANOVA** | F(3,8)=33.30, P≤0.0001 | **Dunnett’s**  WT: Itgb1^-/-^ (2x) | ns (P>0.05) |
| 6 – fig suppl 2 | **ANOVA** | F(3,8)=15.81, P≤0.001 | **Dunnett’s**  WT: Tln1^-/-^  WT: Itgb2^-/-^  WT: Itgb1^-/-^ | ns (P>0.05)  ** (P≤0.01)  ns (P>0.05) |
| 7D | **ANOVA** | F(3,8)=33.94, P≤0.0001 | **Dunnett’s**  WT: Tln1^-/-^  WT: Itgb2^-/-^  WT: Itgb1^-/-^ | ***(P≤0.001)  ns (P>0.05)  ** (P≤0.01) |
| 7 – fig suppl 2 | **ANOVA** | F(3,8)=35.7, P≤0.0001 | **Dunnett’s**  WT: Tln1^-/-^  WT: Itgb2^-/-^  WT: Itgb1^-/-^ | ***(P≤0.001)  ns (P>0.05)  ** (P≤0.01) |
| 8D | **unpaired two-tailed**  ***t* test** | t=4.695, df=4, P=0.0093 | - | ** (P≤0.01) |
