# Supplementary material for: Macrophage network dynamics depend on haptokinesis for optimal local surveillance
Source: eLife. 2022 Mar 28;11:e75354. doi: 10.7554/eLife.75354 (PMC8963880; doi:10.7554/eLife.75354)
Supplement: Transparent reporting form [file elife-75354-transrepform1.pdf]

Westbrook Centre
Milton Road
Cambridge CB4 1YG, UK
P 01223 855340
W elifesciences.org
T @elife

eLife's transparent reporting form

We encourage authors to provide detailed information *within their submission* to facilitate the interpretation and replication of experiments. Authors can upload supporting documentation to indicate the use of appropriate reporting guidelines for health-related research (see EQUATOR Network), life science research (see the BioSharing Information Resource), or the ARRIVE guidelines for reporting work involving animal research. Where applicable, authors should refer to any relevant reporting standards documents in this form.

If you have any questions, please consult our Journal Policies and/or contact us: editorial@elifesciences.org.

Sample-size estimation

- You should state whether an appropriate sample size was computed when the study was being designed
- You should state the statistical method of sample size computation and any required assumptions
- If no explicit power analysis was used, you should describe how you decided what sample (replicate) size (number) to use

Please outline where this information can be found within the submission (e.g., sections or figure legends), or explain why this information doesn't apply to your submission:

Information in "Statistics section" of Materials and Methods.

Sample sizes for technical replicates (i.e. the tracking of randomly chosen migrating cells) in one biological replicate were considered based on the mean and standard deviation of WT macrophage speed during migration. We defined a 30% reduction of mean speed at a power of 0.95 as biologically meaningful effect, determining a sample size of *N*=25. Sample sizes for biological replicates in cell culture experiments (i.e. BMDM cultures generated from different individual mice) aimed for a minimum of mouse donors to reduce the number of laboratory animals. Sample size for animal experimentation was determined according to animal welfare guidelines.

Replicates

- You should report how often each experiment was performed
- You should include a definition of biological versus technical replication
- The data obtained should be provided and sufficient information should be provided to indicate the number of independent biological and/or technical replicates
- If you encountered any outliers, you should describe how these were handled
- Criteria for exclusion/inclusion of data should be clearly stated
- High-throughput sequence data should be uploaded before submission, with a private link for reviewers provided (these are available from both GEO and ArrayExpress)

Please outline where this information can be found within the submission (e.g., sections or figure legends), or explain why this information doesn't apply to your submission:

eLife Sciences Publications, Ltd is a limited liability non-profit non-stock corporation incorporated in the State of Delaware, USA, with company number 5030732, and is registered in the UK with company number FC030576 and branch number BR015634 at the address Westbrook Centre, Milton Road, Cambridge CB4 1YG.

Information in “Statistics section” of Materials and Methods, detailed numbers are provided in the relevant Figure legends and Supplementary Table:

Technical replicates of one biological replicate were designated with “N“, biological replicates were designated with “n“. Reproducibility of the experimental findings was verified using biological replicates, which were performed as independent experiments.

Information on exclusion/inclusion of data in “Image Analysis section” of Materials and Methods:

For 3D matrigel migration analysis: Cells that underwent cell division during the imaging period were excluded.
For 3D chemotaxis experiments: In rare cases, biological replicates were excluded from analysis when macrophages did not respond to the attractant and only very few control cells performed directed migration.
For intravital microscopy: All protrusions from all biological replicates were tracked and the results statistically analysed.

Statistical reporting

- Statistical analysis methods should be described and justified
- Raw data should be presented in figures whenever informative to do so (typically when N per group is less than 10)
- For each experiment, you should identify the statistical tests used, exact values of N, definitions of center, methods of multiple test correction, and dispersion and precision measures (e.g., mean, median, SD, SEM, confidence intervals; and, for the major substantive results, a measure of effect size (e.g., Pearson's r, Cohen's d)
- Report exact p-values wherever possible alongside the summary statistics and 95% confidence intervals. These should be reported for all key questions and not only when the p-value is less than 0.05.

Please outline where this information can be found within the submission (e.g., sections or figure legends), or explain why this information doesn’t apply to your submission:

Information in “Statistics section” of Materials and Methods, detailed numbers are provided in the relevant Figure legends and Supplementary Table.

(For large datasets, or papers with a very large number of statistical tests, you may upload a single table file with tests, Ns, etc., with reference to sections in the manuscript.)

Group allocation

- Indicate how samples were allocated into experimental groups (in the case of clinical studies, please specify allocation to treatment method); if randomization was used, please also state if restricted randomization was applied
- Indicate if masking was used during group allocation, data collection and/or data analysis

Please outline where this information can be found within the submission (e.g., sections or figure legends), or explain why this information doesn’t apply to your submission:

Information in “Statistics section” of Materials and Methods:

Experimental groups were mostly defined by the genotype, or by inhibitor treatment of WT cells. Randomization, masking and blinding was not applied in our study.

Additional data files ("source data")

- We encourage you to upload relevant additional data files, such as numerical data that are represented as a graph in a figure, or as a summary table
- Where provided, these should be in the most useful format, and they can be uploaded as "Source data" files linked to a main figure or table
- Include model definition files including the full list of parameters used
- Include code used for data analysis (e.g., R, MatLab)
- Avoid stating that data files are "available upon request"

Please indicate the figures or tables for which source data files have been provided:

Numerical data for graphs in Figures 1D, 1E, 1G, 1H, 1I, 3D, 3E, 3F, 3G, 4D, 5D, 5E, 5F, 5K, 5L, 5N, 5O, 6C, 6D, 7D and figure supplements are provided as .csv files.

eLife Sciences Publications, Ltd is a limited liability non-profit non-stock corporation incorporated in the State of Delaware, USA, with company number 5030732, and is registered in the UK with company number FC030576 and branch number BR015634 at the address Westbrook Centre, Milton Road, Cambridge CB4 1YG.
